# The risk factors for diabetic peripheral neuropathy: A meta-analysis

**DOI:** 10.1371/journal.pone.0212574

**Published:** 2019-02-20

**Authors:** Xiuxiu Liu, Yuyan Xu, Miaomiao An, Qibing Zeng

**Affiliations:** Key Laboratory of Environmental Pollution Monitoring and Disease Control, Ministry of Education, Department of Toxicology, School of Public Health, Guizhou Medical University, Guiyang, Guizhou, China; Universidad Miguel Hernandez de Elche, SPAIN

## Abstract

Diabetic peripheral neuropathy (DPN), the most common chronic complication of diabetes, has become an important public health crisis worldwide. Given that DPN is extremely difficult to treat, determining its risk factors and controlling it at an early stage is critical to preventing its serious consequences and the burden of social disease. Current studies suggest that the risk factors for diabetic peripheral neuropathy are the duration of diabetes, age, glycosylated hemoglobin A1c (HbA1c), diabetic retinopathy (DR), smoking, and body mass Index (BMI). However, most of the aforementioned studies are cross-sectional, and the sample sizes are very limited, so the strength of causal reasoning is relatively low. The current study systematically evaluated DPN’s influencing factors in patients with type 2 diabetes using evidence-based medicine. Overall, 16 included studies (14 cross-sectional studies and 2 case-control studies including 12,116 cases) that conformed to the present criteria were included in the final analysis. The results suggested that the duration of diabetes (MD 2.5, 95% CI 1.71~3.29), age (MD 4.00, 95% CI 3.05~4.95), HbA1c (MD 0.48, 95% CI 0.33~0.64), and DR (OR 2.34, 95% CI 1.74~3.16) are associated with significantly increased risks of DPN among diabetic patients, while BMI, smoking, total triglyceride (TG), and total cholesterol (TC) did not indicate any risks of increasing DPN. The findings provide a scientific basis for a further understanding of the causes of type 2 diabetes complicated with peripheral neuropathy and the improvement of preventive strategies. The next step is to conduct further high-quality prospective cohort studies to validate this paper’s findings.

## Introduction

Diabetes has become a global public health crisis. The International Diabetes Federation estimated that there were 451 million people (ages 18–99 years) with diabetes worldwide in 2017. Approximately 85%-95% of patients are diagnosed with type 2 diabetes mellitus (T2DM) in high-income countries. The proportion may be higher in other countries with lower incomes[1, 2]. Diabetic peripheral neuropathy (DPN) is the most common cause of neuropathy worldwide. It is estimated to be present in approximately half of those with diabetes, and 10% to 20% have symptoms that are severe enough to warrant treatment[3, 4]. The Toronto Consensus meeting defined typical DPN as asymmetrical and length-dependent sensorimotor polyneuropathy attributed to metabolic and microvessel alterations as a result of a background of long-standing hyperglycaemia and metabolic derangements[5]. A retrospective study based on a large US commercial database indicated that after DPN diagnosis, the annual cost per patient to visit hospitals, emergency rooms (ERs), doctor's offices, and pharmacies increased by 46% and the highest increase (60%) was in hospitalisation costs[6]. The total annual cost of treating DPN and its related complications in all patients in the United States is estimated at $760 million (type 1 diabetes), $10.15 billion (type 2 diabetes), and $10.91 billion (type 1 and type 2 diabetes)[7]. Other research indicated that the financial and health burdens of diabetic foot care in 2010–2011 were estimated at £580 million in England, which represented approximately 0.6% of the National Health Service expenditures at that time[8]. The aforementioned data show that DPN has caused severe challenges to health expenditures globally. Effectively controlling the substantial health expenditures resulting from DPN is a worldwide concern.

Patients with diabetes are extremely difficult to treat once they develop neuropathy. Identifying the modifiable risk factors for the development of neuropathy and effectively controlling them at an early stage is critical for the successful management of diabetes and preventing serious DPN-related consequences (such as ulcer, gangrene, and amputation) and social disease burdens[9, 10]. According to reports in the literature, appropriate interventions can reduce ulcers by 60% and amputations by 85% in those with high-risk diabetic neuropathy[11]. Current studies suggest that risk factors for diabetic peripheral neuropathy include the duration of diabetes[12–18], age[12, 13, 17, 18], HbA1c[14, 16], DR[14, 16, 18], smoking[12, 18, 19], and BMI[12, 14], fasting plasma glucose (FPG)[20, 21], blood urea nitrogen(BUN)21,34, diastolic blood pressure(DBP)21,25 amongst others. However, most of the aforementioned reports are cross-sectional studies, and the sample sizes are very limited, so the strength of causal reasoning is relatively low. The aim of this study is to systematically evaluate the influencing factors of peripheral neuropathy in patients with type 2 diabetes using evidence-based medicine. This report provides a scientific basis for a greater understanding of the causes of type 2 diabetes complicated with peripheral neuropathy and preventive strategies.

## Materials and method

### Literature search

We searched the English databases PubMed, Web of Science, Embase, and the Wiley Online Library and Chinese databases such as China National Knowledge Infrastructure (CNKI), VIP Information, and Wanfang Data to identify relevant citations published between January 2000 and May 2018. We used a combination of search mesh terms related to diabetic peripheral neuropathy (“diabetic peripheral neuropathy” and “diabetic neuropathy”) and risk factors (“risk factor” and “influence factor”). We also reviewed the bibliographies from the citations of relevant articles.

### Selection criteria and process

The inclusion criteria were ① case-control or cohort studies or cross-sectional reports investigating DPN’s risk factors published both domestically and overseas from 2000—to 2018, ② DM and DPN were medically confirmed, and ③ reported outcome measures with odds ratios (ORs) or relative risks (RRs) with 95% confidence intervals.

The exclusion criteria were ① editorials, letters to the editor, review articles, case reports, and animal experimental studies; ② missing primary data; and ③ studies concerning type 1 diabetes.

We found articles that were eligible for further review by initially screening identified titles or abstracts and then conducting a full text review. All of the possibly relevant papers were reviewed independently by two investigators, and disagreements were resolved by discussion or by the third investigator.

### Assessment of methodological quality

We assessed the methodological quality of the included studies based on the Newcastle-Ottawa Scale (NOS) for case-control or cohort studies and the Agency for Healthcare Research and Quality (AHRQ) for cross-sectional reports.

### Data extraction

Abstracted relevant information was obtained from each qualified study using a standardised form. Information regarding the characteristics of the study population, study design, and risk factors was recorded.

The extract prevalence data were ① the number of people with DPN and those who had been tested for DPN and ② risk factor data including the definition of risk factors, ORs, RRs, and corresponding confidence intervals (CIs).

The diagnosis of DM was based on the ADA or WHO diagnostic criteria. The diagnostic criteria of DPN differed domestically and overseas, so the included studies had to list the diagnostic standards.

### Statistical analysis

All of the meta-analyses were performed using Review Manager 5.3 (Cochrane Collaboration, Oxford, UK). The weighted mean differences (WMDs) and odds ratios (ORs) were used to compare continuous and dichotomous variables. All of the results were reported with 95% confidence intervals. We used the random-effects model or the fixed-effects model to assess the pooled risk estimates reported as odds ratios and 95% confidence intervals. Heterogeneity across the studies was assessed statistically using the I-squared (I^2^) test. When I^2^>50%, we used the random-effects model, and when I^2^<50%, we used the fixed-effects model. We used a sensitivity analysis and a subgroup analysis to explore sources of heterogeneity. Publication bias was assessed using the RevMan 5.3 funnel plot procedure.

## Results

### Study identification and selection

**Fig 1:**
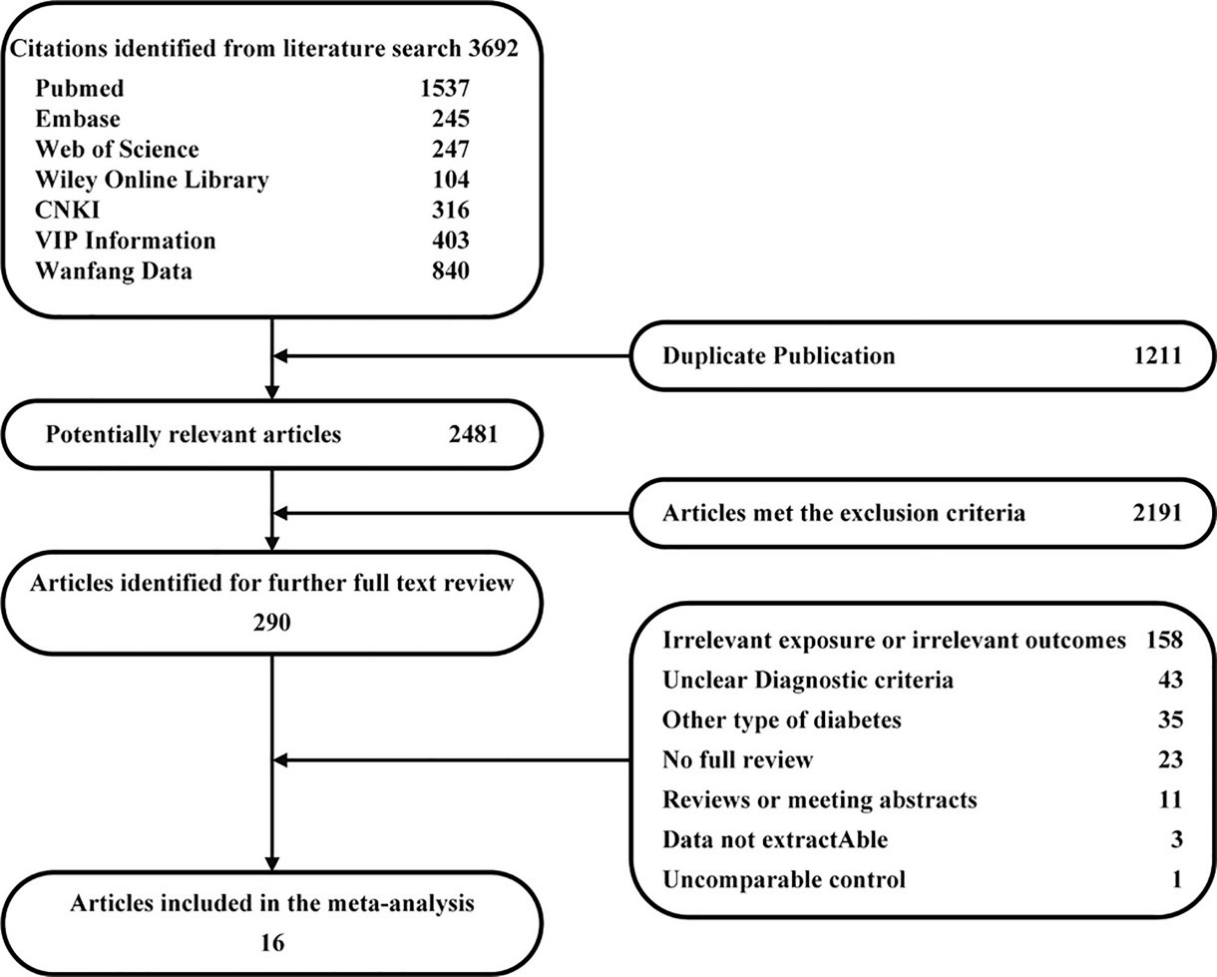
Flowchart of the meta-analysis.

The search strategy identified 3,692 unique citations. After eliminating duplicate literature, 2,481 potentially relevant articles remained. After a second round of screening based on titles and abstracts with the exclusion criteria, 290 articles remained for further evaluation. After a detailed examination, 273 articles were excluded for the reasons shown in Fig 1. Finally, 15 cross-sectional studies and 2 case-control studies were assessed among the 17 included studies, including 12,216 cases [4,205 cases of DPN and 8,011 cases of Non-diabetic peripheral neuropathy (NDPN)] that conformed to the pre-set criteria. These were included in the final analysis. According to the NOS and AHRQ quality standards, the results of all of the studies are shown in Tables 1 and 2. The characteristics of the included studies are shown in Table 3.

**Table 1:** Methodological quality of studies included in the final analysis based on the AHRQ for assessing the quality of cross-sectional study.

Study ID	Difine the source	List criteria	Indicate time	Indicate subjects	Indicate mask	Describe assessment	Explain exclusion	Describe confounding	Hand missing data	Summarize	Follow-up	Total
**Xiaowen Chen[22]**	1	1	1	1	1	1	0	1	0	0	0	7
**Wei Wei[21]**	1	1	1	1	1	0	1	1	0	0	0	7
**Lanying Chen[20]**	1	1	1	1	1	1	0	1	0	0	0	7
**Hui Wan[23]**	1	1	1	1	1	1	0	1	0	0	0	7
**Guangyu He[24]**	1	1	1	1	1	1	0	1	0	0	0	7
**Xiaoqian Chen[25]**	1	1	1	1	1	1	0	1	0	0	0	7
**Bansal, D.[26]**	1	1	1	1	1	1	0	1	0	0	0	7
**Hu, Y.M.[27]**	1	1	1	1	1	1	0	1	0	0	0	7
**Morkrid, K.[28]**	1	1	1	1	1	1	0	1	0	0	0	7
**Shehab, D. [29]**	1	1	1	1	1	1	0	1	0	0	0	7
**Su, J. B.[30]**	1	1	1	1	1	1	1	1	0	0	0	8
**Juan Shen[31]**	1	1	1	1	1	1	0	1	0	0	0	7
**Katulanda, P.[32]**	1	1	1	1	1	1	0	1	1	1	0	9
**Fangzhou Hu[33]**	1	1	1	1	1	1	0	1	0	0	0	7

**Table 2:** Methodological quality of studies included in the final analysis based on the NOS for assessing the quality of case-control study.

Study ID	Object of study				Comparability between groups	Exposure measurement			Score
	Case identification	Case representation	Control selection	Control identification		Determination of exposure factors	Methods for determining exposure factors	No response rates	
**Feng Xu[34]**	1	1	1	1	2	0	1	0	7
**Ybarra-Munoz, J.[35]**	1	1	1	1	2	1	1	0	8

**Table 3:** Characteristics of included studies.

Study ID	Published Year	Study country	Study design	DPN/NDPN	The diagnosis of DPN	Influencing factor	Study
**Xiaowen Chen[22]**	2014	China	Cross-section	90/115	TCSS	age, duration of diabetes, DR, serum creatinine (Scr), Ua1b/Cr, DBP, BUN, weight	hospital
**Wei Wei[21]**	2017	China	Cross-section	141/286	Diagnostic criteria for diabetic peripheral neuropathy	age, smoking, DR, HbA1C, fasting plasma glucose (FPG)	hospital
**Lanying Chen[20]**	2014	China	Cross-section	102/117	Related research	duration of diabetes, HbA1C, FPG, 2hPG, 2hC-P, Scr, Cystatin C (Cys-C)	hospital
**Hui Wan[23]**	2017	China	Cross-section	264/413	Practical endocrinology	age, HbA1C, fasting c-peptide (FC-P), 1 hours c-peptide (1hC-P), 2 hours c-peptide (2hC-P),	hospital
**Guangyu He[24]**	2014	China	Cross-section	71/42	Diagnostic criteria for diabetic peripheral neuropathy	age, duration of diabetes, high-density lipoprotein cholesterol (HDL-C)	hospital
**Xiaoqian Chen[25]**	2011	China	Cross-section	1308/1658	DNS	age, HbA1C, duration of diabetes, FPG, PG, waist-to-hip ratio, systolic blood pressure (SBP)	hospital
**Bansal, D.[26]**	2014	India	Cross-section	586/1420	NDS	age, duration of diabetes, smoking, BMI, HbA1C, alcohol, socioeconomic status, hypertension, low- density lipoprotein cholesterol (LDL-C), HDL-C, TC, dyslipidemia, nephropathy	hospital
**Hu, Y.M.[27]**	2018	China	Cross-section	197/785	Symptoms, nerve conduction test	duration of diabetes, HbA1C, insulin injections, hypertension, homeostasis model assessment of insulin resistance (HOMA-IR), mean amplitude of glycaemic excursions (MAGE), mean of daily differences (MODD), standard deviation of glucose (SD) and 24-h mean glucose (24-h MG)	hospital
**Morkrid, K.[28]**	2010	Bangladesh	Cross-section	58/236	NSS	age, duration of diabetes, HbA1C, low protein intake, oral treatment, insulin treatment, DBP	hospital
**Shehab, D. [29]**	2012	Kuwait	Cross-section	87/123	NSS/NDS	age, duration of diabetes, HbA1C, Vitamin D, LDL-C	Population
**Su, J. B.[30]**	2018	China	Cross-section	102/461	NDS	age, duration of diabetes, insulin resistance index (HOMA-IR), initial HbA1c, urinary albumin-to-creatinine ratio (UACR), mean of HbA1c (M-HbA1c), coefficient of variation of HbA1c (CV-HbA1c)	Hospital
**Juan Shen[31]**	2016	China	Cross-section	397/734	NCV results	duration of diabetes, HbA1C, BUN, creatinine (Cr), glycosylated albumin (GA), 30-min postprandial C-peptide (30-min PCP), 120-min postprandial C-peptide (120-min PCP)	Hospital
**Katulanda, P.[32]**	2012	Sri Lanka	Cross-section	127/401	DNS.TCSS	duration of diabetes, TC, smoking, DR, BMI, gender, sector of residence, household monthly income, height, foot ulcers, drug treatment, Insulin	Community
**Ybarra-Munoz, J.[35]**	2016	Spain	Nested case-control	49/218	NSS	age, duration of diabetes, CVD (cardiovascular disease), LDL-C	Population
**Feng Xu[34]**	2014	China	Case-contorl	45/45	TCSS	BMI, TC, LDL-C, standard deviation of blood glucose (SDBG), MODD, MAGE	hospital
**Fangzhou Hu[33]**	2018	China	Cross-section	119/121	Guidelines for prevention and treatment of type 2 diabetes in China (2013 edition)	age, duration of diabetes, DR, Fasting Blood Glucose (FBG), 2hC-P, free fatty acid (FFA), BUN, coronary artery heart disease (CHD)	hospital

### Risk of bias assessment

Through the literature searches, the included reports were mainly cross-sectional and case-control studies, with no cohort studies. Two cross-sectional studies explained any patients excluded from the analyses, and one summarised the patient response rates. The cross-sectional studies were subject to further follow-up and more case-control studies were expected to be included. The DPN diagnostic standards of the included studies differed due to varying pathophysiologies resulting from the clinical manifestations and differences in mechanisms.

### Meta-analysis results

(1) Duration of diabetes and risk of DPN

**Fig 2:**
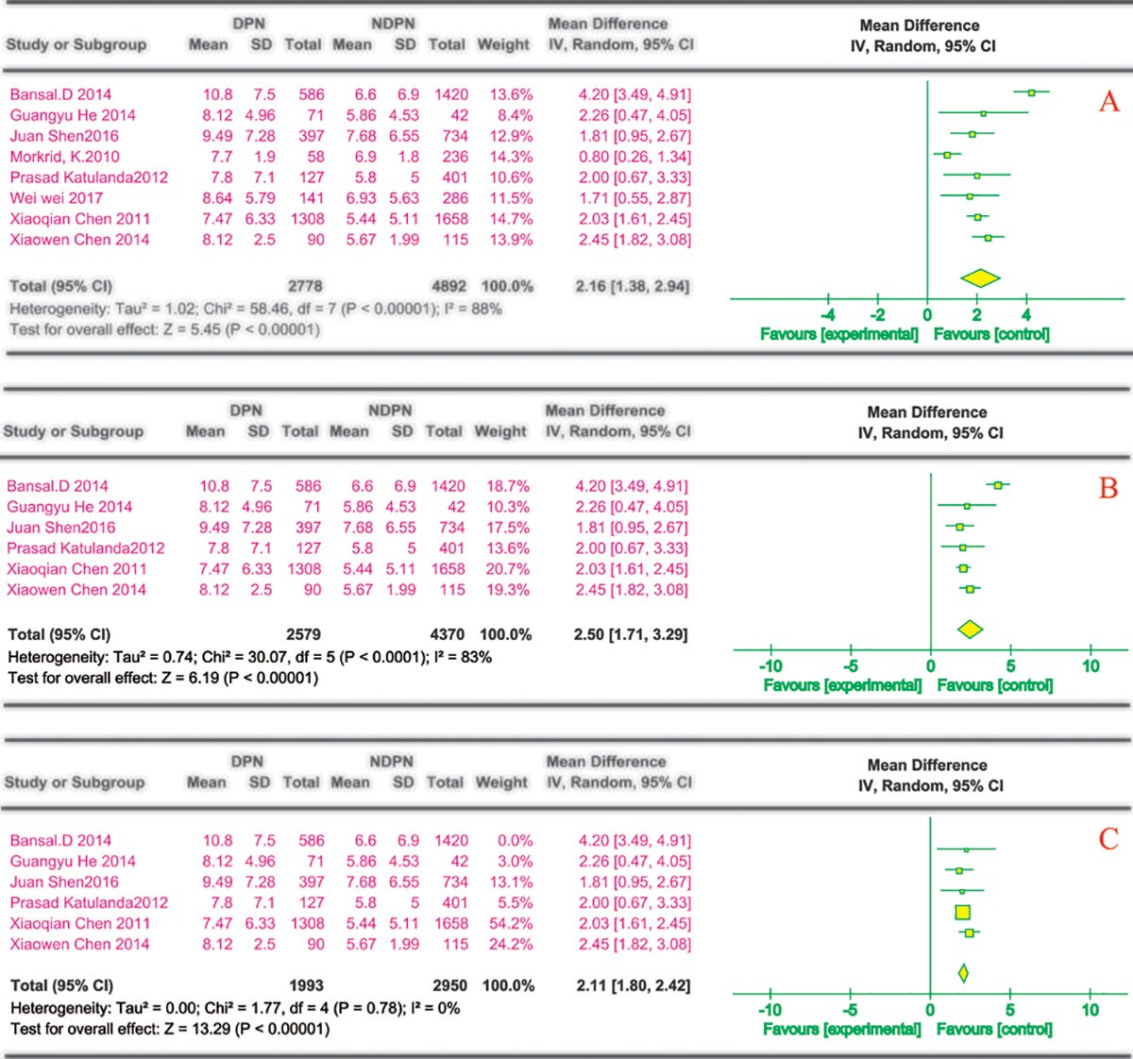
Duration of diabetes and risk of DPN. The summary mean difference was calculated using a random-effects model. The mean difference and 95% CI for each study and the final combined results are displayed numerically on the left and graphically as a forest plot on the right. A and B are based on univariate analysis and multivariate analysis data, respectively. The sensitivity analysis results are shown in C.

For DPN risk, 15 reports described the effects of the course of the disease, 13 of which were cross-sectional studies and two of which were case-control studies. Only reports that described the data in terms of mean (±SD) were included. Among the 13 cross-sectional studies, 8 were included. Three of these reports described the results with the median and two described the classification variables for the duration of diabetes. The frequency of disease duration as a categorical variable was provided in both cases-control studies. Eight cross-sectional studies[21, 22, 25, 28, 29, 31, 32, 36] describing the risk factors for DPN in those with D2M via a univariate analysis showed significant differences in the duration of diabetes (MD 2.16, 95% CI 1.38~2.94) (Fig 2A). Using a multivariate analysis, six cross-sectional studies[22, 25, 29, 31, 32, 36] indicated significant changes in the duration of diabetes, with MD 2.50 and the pooled 95% CI ranging from 1.39 to 1.47 (Fig 2B). The results of variable analysis and multivariate analysis are highly heterogeneous (I^2^ *=* 88%, 83%) (Fig 2A and 2B). Therefore, the random-effects model was used to pool the results. A sensitivity analysis omitting one study in each turn showed no substantial changes in the results with pooled ORs, and when one study[36] of the duration of diabetes was removed from the multivariate analysis, the heterogeneity decreased to 0% (Fig 2C).

(2) Age and risk of DPN

**Fig 3:**
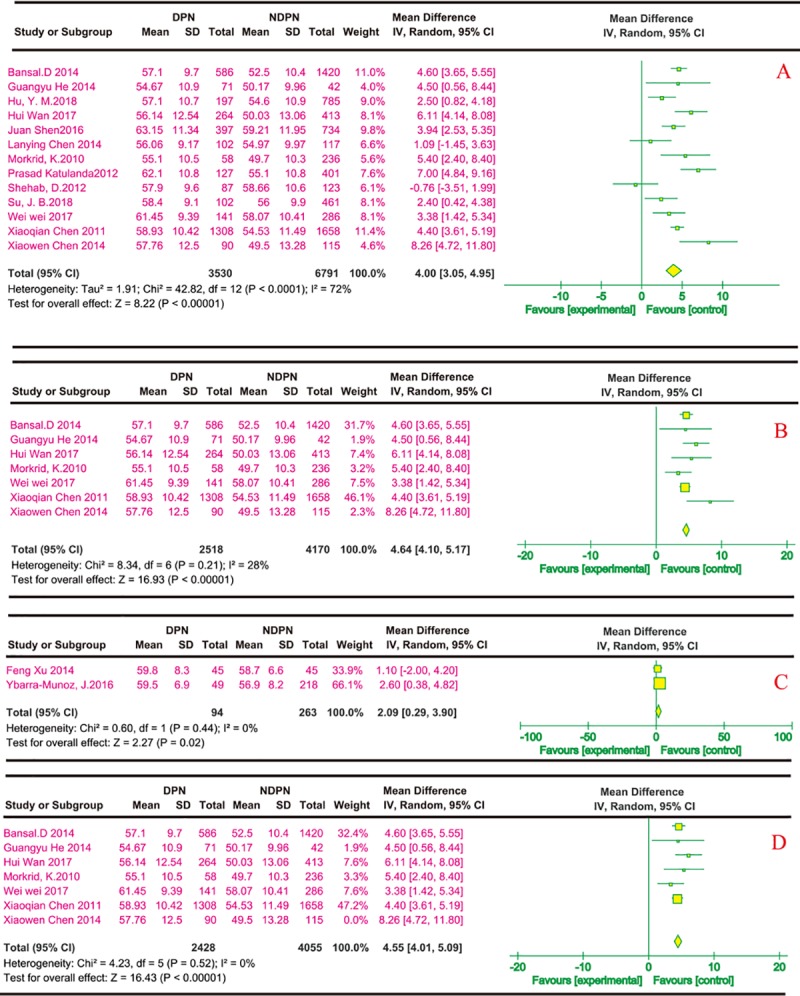
Age and risk of DPN. The summary mean difference was calculated using a random-effects model. The mean difference and 95% CI for each study and the final combined results are displayed numerically on the left and graphically as a forest plot on the right. A and B are based on univariate analysis and multivariate analysis data for cross-sectional studies, respectively. C showed significant differences in age in the case-control studies. The sensitivity analysis results are shown in D.

All of the reports described the effects of age on diabetic peripheral neuropathy; 14 were cross-sectional studies and two were case-control studies. Of these reports, only those that described the data in terms of mean (±SD) were included, so 13 cross-sectional and two case-control studies were included. Fig 3A shows that the effects of age on DPN were reported in 13 cross-sectional studies[20–25, 27–32, 36], which had significant differences in age (MD 4.00, 95% CI 3.05~4.95) via the univariate analysis. In the multivariate analysis, six cross-sectional studies[21–25, 28, 36] showed significant differences in age, with MD 4.64 and 95% CI ranging from 4.10 to 5.17 (Fig 3B). Two case-control studies showed significant differences in age, but the MD was lower than the cross-sectional study at 2.09 and the 95% CI ranging from 0.29 to 3.90 (Fig 3C). A sensitivity analysis of the univariate analysis indicated there was no change in the significance of the outcomes. The sensitivity analysis of the multivariate analysis showed that the degree of heterogeneity decreased slightly regarding age after removing a study[22] (Fig 3D).

(3) HbA1c and risk of DPN

**Fig 4:**
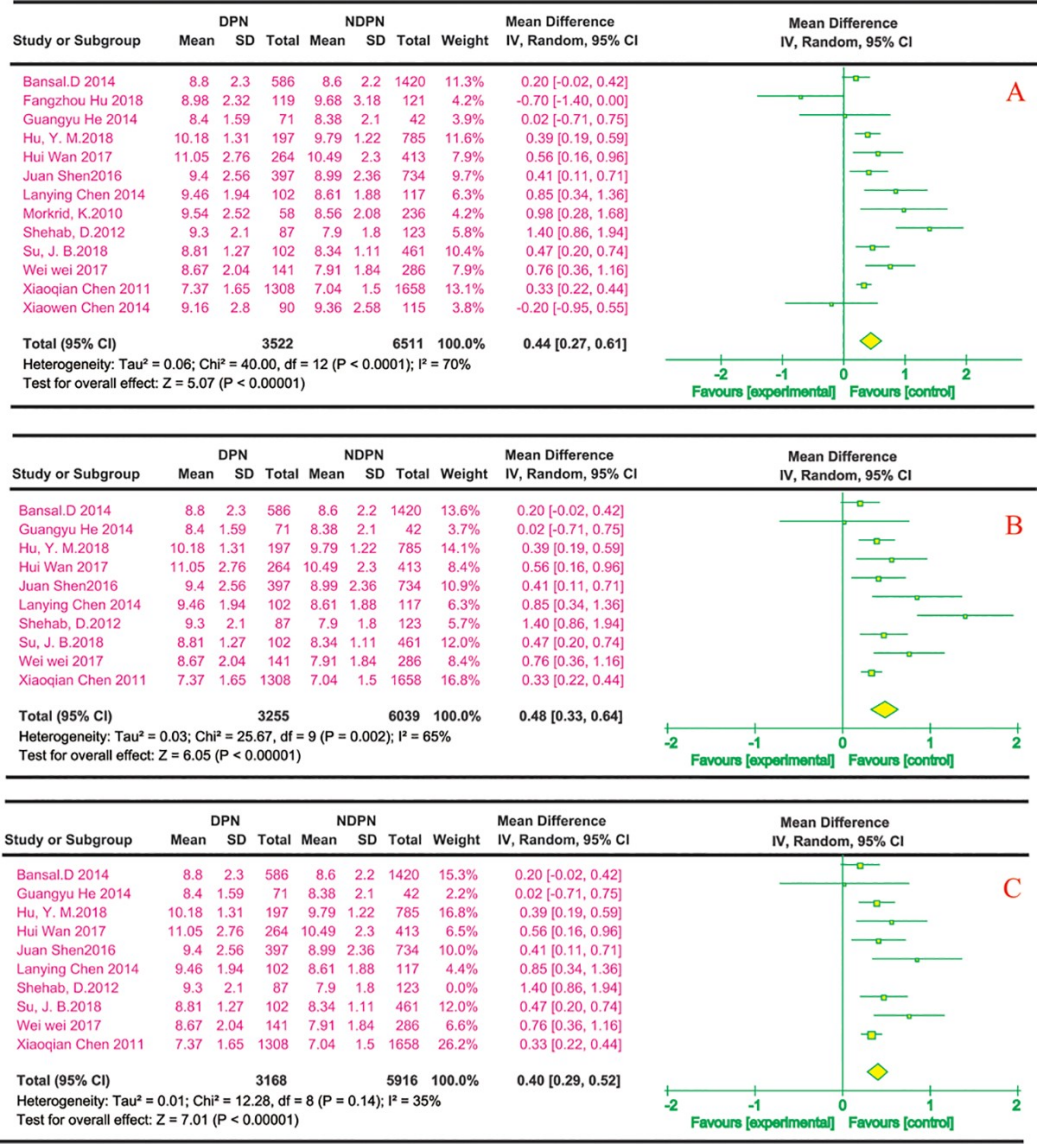
HbA1c and risk of DPN. The summary mean difference was calculated using a random-effects model. The mean difference and 95% CI for each study and the final combined results are displayed numerically on the left and graphically as a forest plot on the right. A and B are based on univariate analysis and multivariate analysis data, respectively. The sensitivity analysis results are shown in C.

Thirteen reports described the effects of HbA1c on DPN; all were cross-sectional. Most of the studies were conducted in China (n = 10), and the others (n = 3) were from India, Bangladesh, and Kuwait. Fig 4A shows that the effects of HbA1c on DPN in 13 studies[20–25, 27–31, 33, 36] had significant differences via a univariate analysis (MD 0.44, 95% CI 0.27~0.61). For the multivariate analysis, 10 cross-sectional studies[20, 21, 23–25, 27, 29–31, 36] indicated significant differences in HbA1c between DPN and NDPN (MD 0.48, 95% CI 0.33~0.64) (Fig 4B). A sensitivity analysis of the univariate analysis showed there was no change in the significance of the outcomes. The sensitivity analysis of the multivariate analysis showed the heterogeneity decreased when a study was deleted[29] (Fig 4C).

(4) DR and risk of DPN

**Fig 5:**
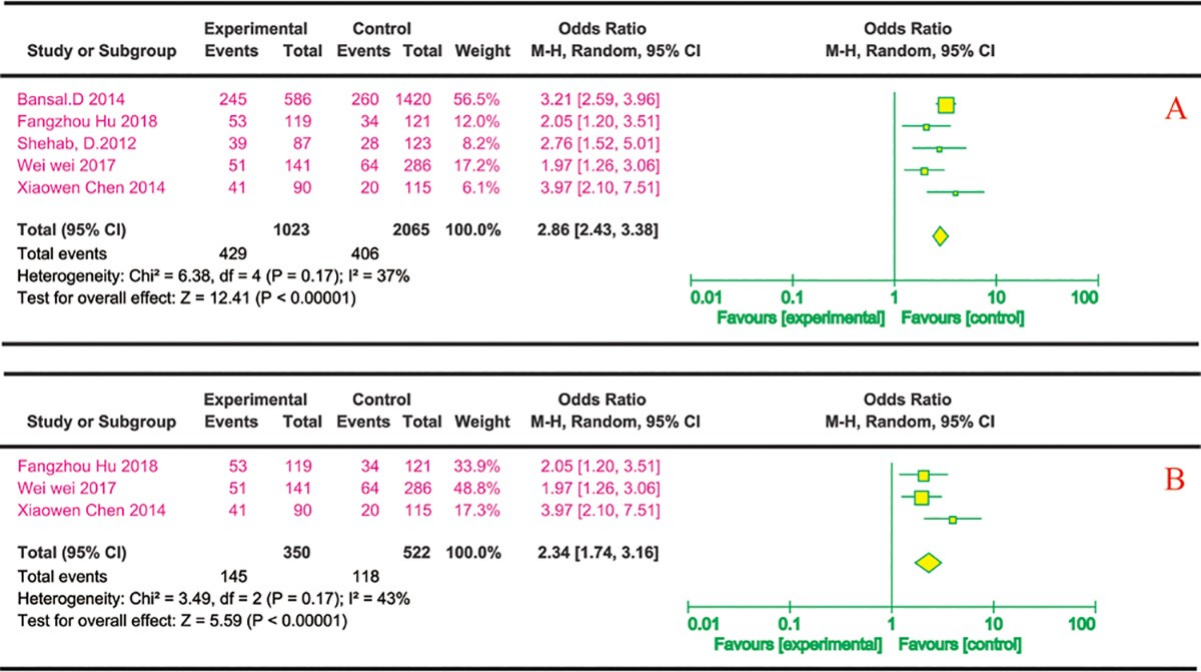
DR and risk of DPN. The summary odds ratio was calculated using a fixed-effects model. The odds ratio and 95% CI for each study and the final combined results are displayed numerically on the left and graphically as a forest plot on the right. A and B are based on univariate analysis and multivariate analysis data, respectively.

Five studies described the effects of DR on DPN, all of which were cross-sectional. Three were from China, one was from India, and one was from Kuwait. Each study described the number of people with DR in both the case group and the control group. Five cross-sectional studies[21, 22, 29, 33, 36] indicated that diabetic DR significantly increased the risk of DPN via univariate analysis (OR 2.86 95% CI 2.43~3.38) (Fig 5A). Concomitantly, for the multivariate analysis, three cross-sectional studies[21, 22, 33] showed similar results, and diabetes DR significantly increased DPN risk (OR 2.34, 95% CI: 1.74~3.16) (Fig 5B).

(5) Smoking and risk of DPN

**Fig 6:**
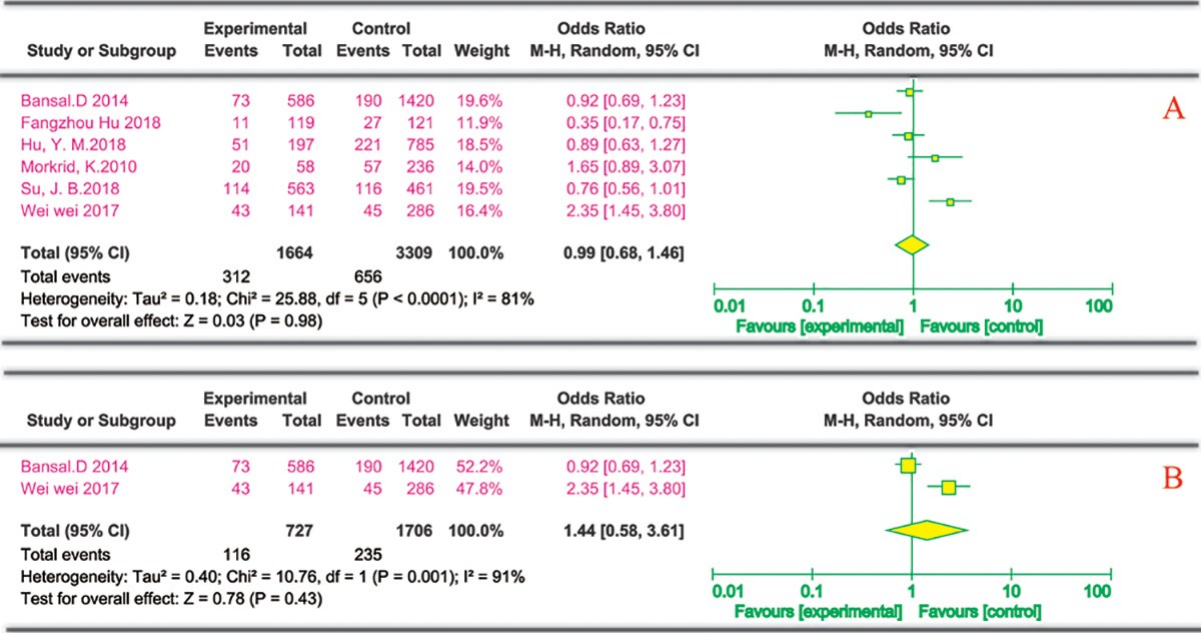
Smoking and risk of DPN. The summary odds ratio was calculated using a random-effects model. The odds ratio and 95% CI for each study and the final combined results are displayed numerically on the left and graphically as a forest plot on the right. A and B are based on univariate analysis and multivariate analysis data, respectively.

Six cross-sectional studies described the effects of smoking on DPN. Four were from China and the others were conducted in India and Bangladesh. Each study described the number of people with smoking in both case groups and the control group. Via univariate analysis, six cross-sectional studies[21, 27, 28, 30, 33, 36] found no significant differences in smoking between the experimental group and the control group (Fig 6A). Fig 6B shows that smoking does not increase the risk of DPN in a multivariate analysis based on the two cross-sectional studies[21, 36].

(6) BMI and risk of DPN

**Fig 7:**
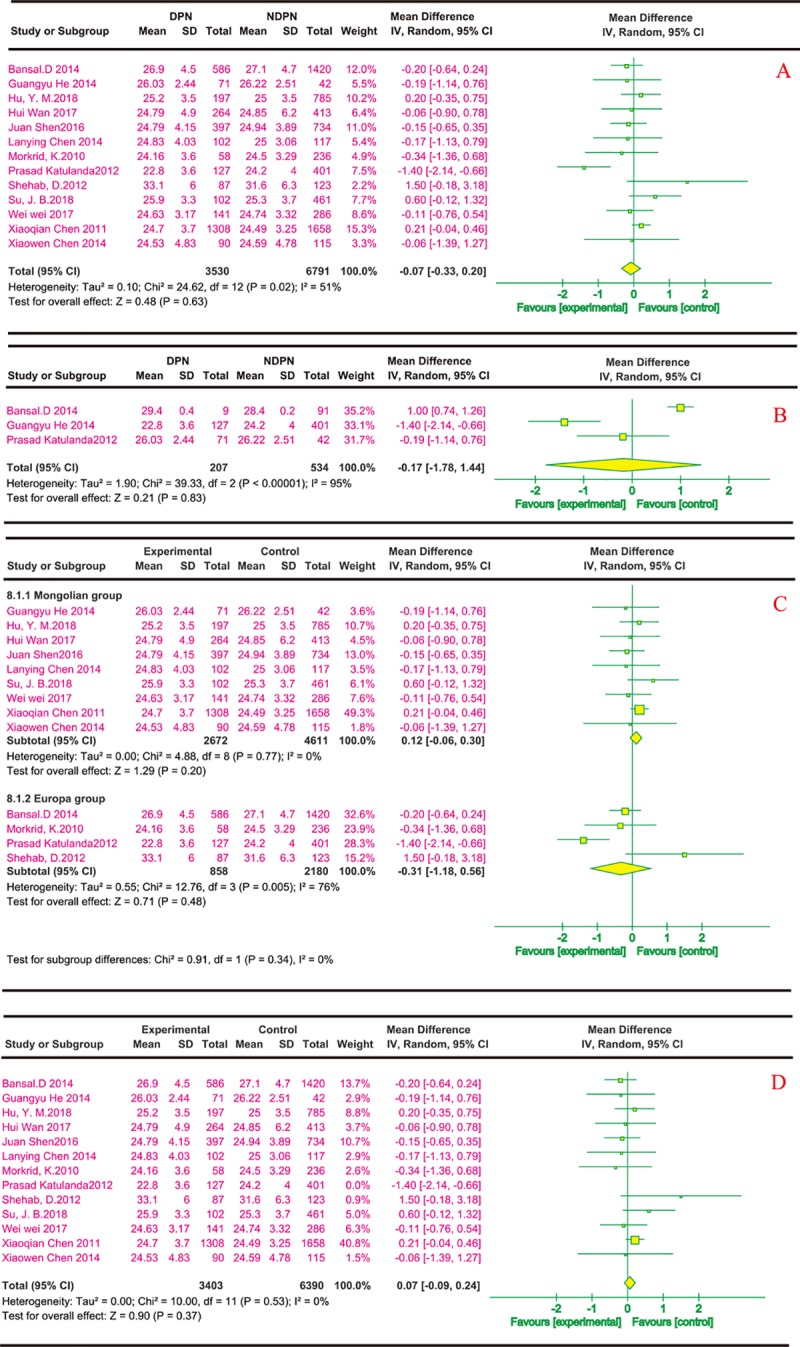
BMI and risk of DPN. The summary mean difference was calculated using a random-effects model. The mean difference and 95% CI for each study and the final combined results are displayed numerically on the left and graphically as a forest plot on the right. A and B are based on univariate analysis and multivariate analysis data, respectively. A subgroup analysis was conducted on the basis of ethnicity; the results are shown in C. The sensitivity analysis results are shown in D.

Thirteen reports described the effects of BMI on DPN; all were cross-sectional. Most [20–25, 27, 30, 31] were conducted in China (n = 9), and the others[28, 29, 32, 36] (n = 4) were from India, Bangladesh, Kuwait, the US, and Sri Lanka. Fig 7A and 7B show that the risk of DPN did not increase significantly with the growth of BMI. In this study, the subgroup analysis was conducted on the basis of ethnicity. Fig 7C showed that the heterogeneity in the European group was high, but there was no heterogeneity in the Mongolian group. Neither of the two subgroups found that BMI can affect DPN. A sensitivity analysis removing one study in each turn showed no substantial changes in the results, but showed the heterogeneity decreased to 0% when a study[32] was removed (Fig 7D).

(7) TC and risk of DPN

**Fig 8:**
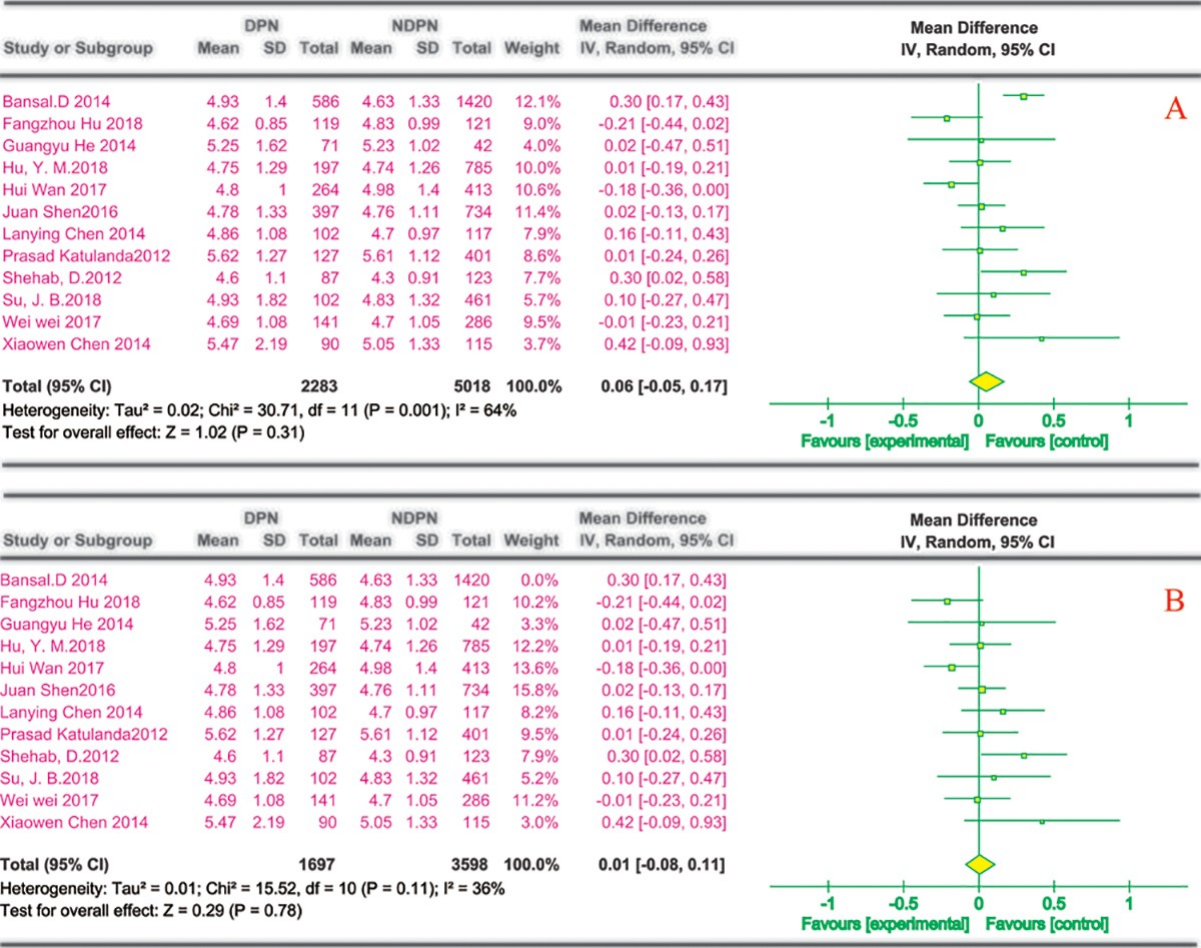
TC and risk of DPN. The summary mean difference was calculated using a random-effects model. The mean difference and 95% CI for each study and the final combined results are displayed numerically on the left and graphically as a forest plot on the right. A and B are based on univariate analysis and multivariate analysis data, respectively.

Twelve reports described the effects of TC on DPN; all were cross-sectional studies by univariate analysis. Most were conducted in China (n = 8), and the others (n = 4) were from India, Kuwait, the US, and Sri Lanka. All of the studies[20–24, 27, 29–33, 36] showed no significant differences in TC between the DPN and NDPN groups (Fig 8A). A sensitivity analysis omitting one study in each turn showed no substantial changes in the results, and when one study[36] was removed, the heterogeneity decreased to 36% (Fig 8B).

(8) TG and risk of DPN

**Fig 9:**
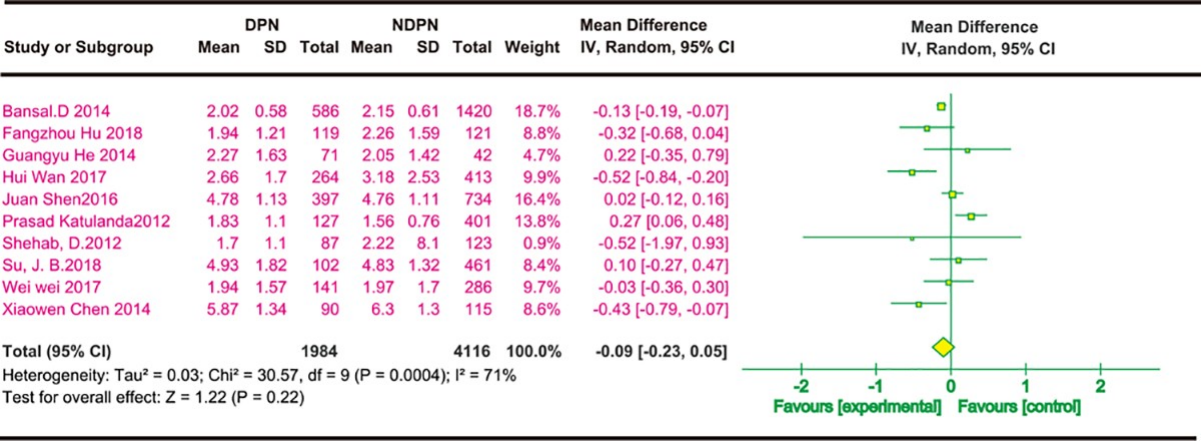
TG and risk of DPN. The summary mean difference was calculated using a random-effects model. The mean difference and 95% CI for each study and the final combined results are displayed numerically on the left and graphically as a forest plot on the right.

Ten reports described the effects of TG on DPN; all were cross-sectional. Most of these studies were conducted in China (n = 7), and the others (n = 3) were from India, Kuwait, the US, and Sri Lanka. In these reports, only those that described the data in terms of mean (±SD) were included. Fig 9 shows that the effects of TG on DPN were conducted in 10 cross-sectional studies[21–24, 29–33, 36], which found no significant differences in TG between the DPN and NDPN groups.

### Publication bias

Risk factors included in no less than 10 studies were analysed for publication bias. All funnel plots using a random-effects model occurred in the studies with high heterogeneity that exceeded 50%. Visual inspection of the funnel plots revealed moderate asymmetry for age, HbA1c, BMI, TC, and TG (S1 Fig).

## Discussion

DPN is often a hidden and gradual process, and the severity of its pathological changes is often inconsistent with the appearance and severity of symptoms[11]. The characteristic clinical manifestation is neuropathic pain (a feeling of burning, tingling electric, sharp, and shooting pain)[26], Subsequently, foot ulceration and amputations may occur[37]. Many patients with painful DPN also reported the interference of pain on their daily activities, such as sleep, enjoyment of life, recreational activities, mobility, normal work, and social activity[38]. A study of 265 patients found that 80% of the DPN patients had depression (27.8%) and anxiety disorders (26.7%)[26]. DPN’s hazards are obvious, but there is no special treatment for DPN in clinics at present, so its early prevention is of great significance.

DPN’s prevalence increases with the duration of the disease[39]. The results of this study showed that the duration of disease had a statistically significant effect on the complications of peripheral neuropathy in patients with type 2 diabetes. This is consistent with previous research results[12–18] suggesting that early screening for primary disease is important to prevent and delay the occurrence of DPN. The results of univariate and multivariate analyses were highly heterogeneous. Therefore, the random-effects model was used to pool the results. A sensitivity analysis omitting one study in each turn showed no substantial changes in the results via a univariate analysis. When one study of the duration of diabetes was removed from the multivariate analysis, the heterogeneity decreased to 0%. It suggested that this article was the main cause of high heterogeneity. Bansal et al.[36] reported a higher OR than other studies, which may have been attributed to the fact that they chose newly diagnosed type 2 diabetics as the control subjects.

Age as a risk factor for many diseases has been confirmed in previous research[12, 13, 17, 18] into DPN and this study. The combined MD values of age shown in case-control studies were larger than those in cross-sectional studies. It is possible that the results of the case-control study were biased due to too little literature. Age was considered a stable exposure factor. The cross-sectional studies were similar to the case-control studies in the strength of causal argumentation. Therefore, we believe the results of the cross-sectional study were more accurate. The sensitivity analysis of age of the multivariate analysis showed that the degree of heterogeneity decreased slightly after a study was removed[22]. An analysis of the reason may be the study used TCSS scores as diagnostic criteria that contributed to some DPN patients without obvious symptoms and signs were missed diagnoses.

Glycated haemoglobin is a product of haemoglobin in the red blood cells combined with sugars in the serum, composed of HbA1a, HbA1b, and HbA1c, of which HbA1c accounts for approximately 70%. Its structure is stable, so it is used as a monitoring indicator for diabetes control. HbA1c as a risk factor for DPN has been confirmed by some studies[14, 16]. According to research, patients with type 2 DM and HbA1c≥7.0% exhibited an increased risk of DPN, demonstrating a linear relationship[16]. In this study, there were significant differences in HbA1c between the DPN and NDPN groups. It suggests that the early control of HBA1C levels can significantly reduce DPN risk. Relevant studies have shown that BMI can affect HbA1c levels[40]. A sensitivity analysis of a multivariate analysis found that heterogeneity decreased when a study was deleted[29]. In combination with the BMI results in this literature, the difference between the DPN and NDPN groups was higher than in other studies, suggesting that high BMI differences may affect HBA1c levels and thus exhibit higher heterogeneity.

Some studies[12, 14] suggested a positive relationship between BMI and DPN, and we find no association between BMI and DPN both in a random-effects model since the heterogeneity is up to 51%, which is exceeds 50%. However, considering that the included study population is from different countries and involves different races, we believe that further studies are needed to support this conclusion. A sensitivity analysis showed that the degree of heterogeneity decreased to 0% after a study [32] was removed, but the result still showed no association between BMI and DPN. That may be because the study was conducted in a different population, and it did it in community. A subgroup analysis was conducted on the basis of ethnicity. According to the results of a subgroup analysis, there was heterogeneity in the European group, but not in the Mongolian group and the result showed showed no significant differences with DPN in the Mongolian group. This may be related to the European group including a wide range of ethnically diverse countries. In future studies, a larger subgroup analysis based on ethnicity should be used to explain these different findings.

Previous research verified the risk factors of peripheral neuropathy in patients who smoked[12, 18, 19], but in this study, the effect of smoking was not statistically significant. This may be because the literature included herein was related to cross-sectional studies and there were few on risk factors. Therefore, to further explore the relationship between smoking and DPN, higher quality analytical or experimental studies should be conducted.

Lipid composition is very complex. In addition to triglycerides, they also include cholesterol, phospholipids, fatty acids, and a small amount of other lipids. Studies showed that TC[12] and TG[12, 16] is a risk factor for DPN. However, in this study, TC and TG showed no significant differences with DPN. This may be due to the fact that most of the subjects in this study had dyslipidaemia and the long-term use of statins, so the lipid levels did not represent its actual level. In addition, a high heterogeneity of TC was found in the sensitivity analysis. this may be due to literature publication bias, and the forest map results provide substantial proof. The choice of subjects in each study was different, and the research fields also varied; there were also many confounding factors, so no subgroup analysis was conducted.

In addition, the currently reported risk factors for diabetes complicated with peripheral neuropathy include vitamin D[41–43] c-peptide[31], hyperlipidemia[42], alcohol intake[44], hyperglycemia[41, 42], LDL-C[29, 35, 36], BUN[22, 33] amongst others, except the duration of diabetes, age, HbA1c, DR, smoking, BMI, TC, TG. These risk factors play an important role in better explaining the causes of diabetes complicated with peripheral neuropathy. We will further explore these risk factors in future.

## Conclusions

The results herein suggested that the duration of diabetes, age, HbA1c, and DR are associated with significantly increased risks of DPN among diabetic patients, while BMI, smoking, TG, and TC did not indicate a risk of increasing DPN. The findings provide a scientific basis for a further understanding of the causes of type 2 diabetes complicated with peripheral neuropathy and the results of preventive strategies.

This research also has some shortcomings. First, most of the research types were cross-sectional studies, and the strength of causal reasoning was relatively low. Second, the included studies were basically consistent with the diagnosis of type 2 diabetes, but the diagnostic criteria for DPN were not completely consistent with the inclusion criteria of the study subjects. Third, the included literature covered a wide range of areas, but ethnic, economic, and healthcare levels were not considered. Finally, there is publication bias in this paper, so further studies are needed to verify our conclusions. At present, there are few studies on risk factors for DPN in patients with type 2 diabetes; high-quality prospective cohort studies are necessary to elucidate trends in the future.

## Supporting information

S1 Fig Funnel plot of the risk factors for DPN. Risk factors included in no less than 10 studies were analysed for publication bias. All funnel plots used a random-effects model for studies with high heterogeneity that exceeded 50%. Visual inspection of the funnel plot revealed moderate asymmetry for age (A), HbA1c (B and C), BMI (D), TC (E), and TG (F).
(TIF)

S1 File PRISMA checklist. (DOC)
