# Supplementary figures and images for: The risk factors for diabetic peripheral neuropathy: A meta-analysis
Source: PLoS One. 2019 Feb 20;14(2):e0212574. doi: 10.1371/journal.pone.0212574 (PMC6382168; doi:10.1371/journal.pone.0212574)

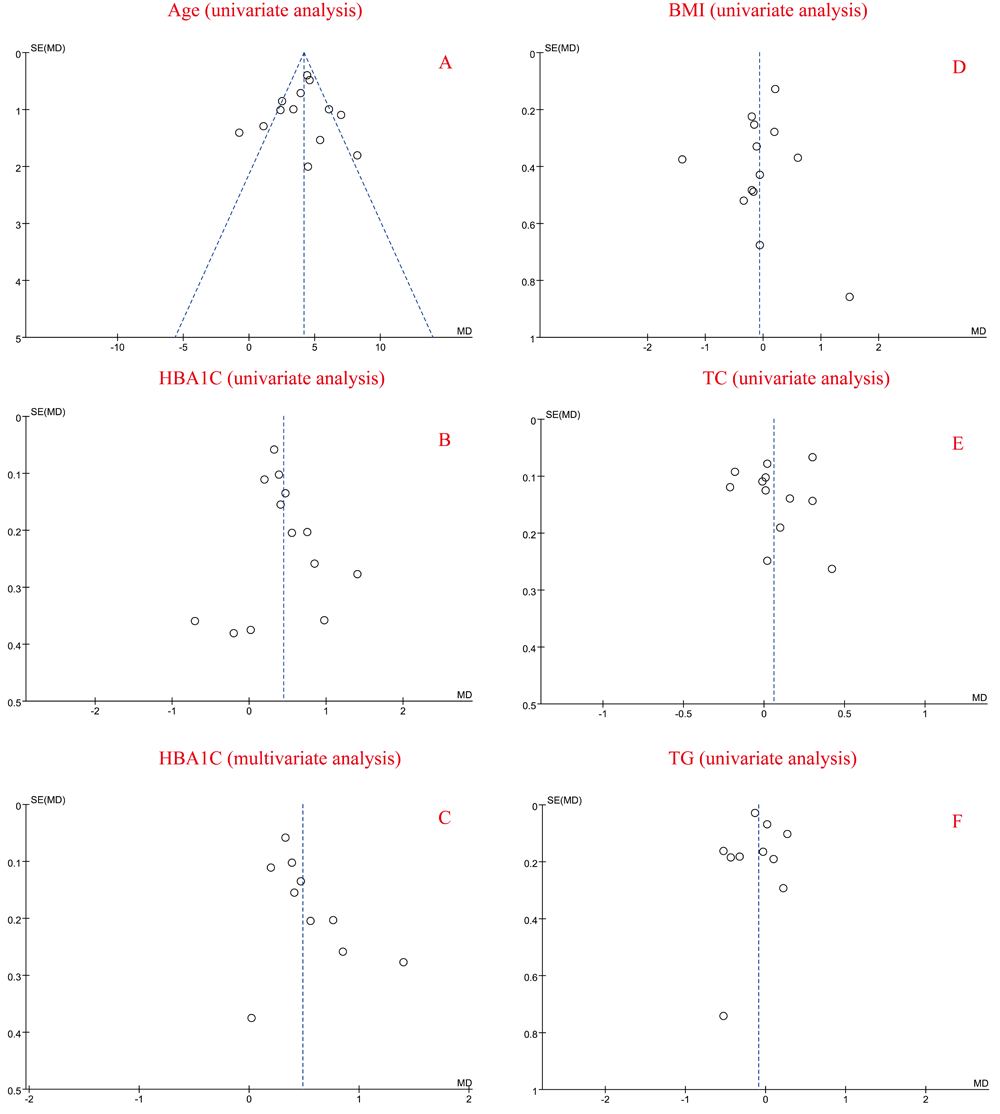

Supplement: S1 Fig — Risk factors included in no less than 10 studies were analysed for publication bias. All funnel plots used a random-effects model for studies with high heterogeneity that exceeded 50%. Visual inspection of the funnel plot revealed moderate asymmetry for age (A), HbA1c (B and C), BMI (D), TC (E), and TG (F). (TIF) [file pone.0212574.s001.tif]
